# Marital Satisfaction, Family Functioning, and Children’s Mental Health—The Effect of Parental Co-Viewing

**DOI:** 10.3390/children9020216

**Published:** 2022-02-06

**Authors:** Xianxian Du, Mengjiao Liang, Weiqi Mu, Fugui Li, Siying Li, Xue Li, Jie Xu, Kexin Wang, Mingjie Zhou

**Affiliations:** 1 CAS Key Laboratory of Mental Health, Institute of Psychology, Chinese Academy of Sciences, Beijing 100101, China; tinadu2009@sina.com (X.D.); Liangmj@clcn.net.cn (M.L.); muwq@psych.ac.cn (W.M.); lifg@psych.ac.cn (F.L.); lisy@psych.ac.cn (S.L.); lixue@psych.ac.cn (X.L.); 2 Department of Psychology, University of Chinese Academy of Sciences, Beijing 100049, China; 3 College of Education, Anyang Normal University, Anyang 455000, China; xj@aynu.edu.cn; 4 College of Media and International Culture, Zhejiang University, Hangzhou 310007, China

**Keywords:** marital satisfaction, parent-child co-viewing, family functioning, child’s mental health

## Abstract

Using 318 household data concerning children during middle childhood (8–13), we examined the association among each parent’s marital satisfaction, the child’s perceived family functioning, and the child’s anxiety and depression. Second, we investigated whether the other parent could buffer or facilitate this chain effect by co-viewing programs via various devices with their child so as to improve the child’s perceived family functioning. Results verified the mediation effect that maternal marital satisfaction was positively associated with the child’s perceived family functioning, which, in turn, decreased the child’s depression. In addition, the results indicated that increased paternal co-viewing enhanced the positive association between maternal marital satisfaction and the child’s perceived family functioning and then decreased the child’s depression. The theoretical and practical implications are discussed within the framework of family systems theory, parental media interventions, and the different roles of the father and mother in family functionality.

## 1. Introduction

During childhood, mental health problems are major concerns for the family and the entire society. The presence of severe mental health problems at this stage sets obstacles for adolescent development [1] and is considered a precursor for mental health problems in adulthood [2]. Children’s mental health problems can often be traced to the dysfunctions of the family and further to the quality of the parent’s marriage because the marital system plays a fundamental role in the entire family. Although research has substantiated the association between marital satisfaction and family functioning [3,4] and the association between family functioning and the child’s mental health problem [5], no empirical research has explicitly investigated the underlying mechanism among marital satisfaction, family functioning, and children’s anxiety and depression.

In addition, the past two decades have witnessed the fact that digital technologies (e.g., the internet, mobile devices) have become fixtures in children’s lives, and media use is weaving densely into the fabric of parent–child interaction. Some scholars are worried that the ubiquity of media is pulling families apart [6], while others believe that media has the potential to enhance family functioning [7]. Among various types of media use in the family context, parent–child media co-use becomes a centrality in research, following the call for encouraging parents to co-use media with children by the American Academy of Pediatrics [8]. Particularly, according to a study based on a representative US sample, approximately one-third of parents reported co-use media (e.g., TV, tablets, and smartphones) most of the time or even all the time [9].

While some studies have investigated the influences of media co-use on children and families [9], much still remains unknown about the influences of such co-use on interactions among family members. For instance, despite media co-use likely facilitating interactions among family members, little is known about how much of a positive impact media co-use may work together with the marital system. With the development of technologies and mobile devices, the most frequent co-use technology observed, TV co-viewing, has expanded to various digital devices, such as tablets and smartphones. Therefore, we used parental co-viewing via these devices as the indicator of parental–child media co-use. The current research aims to investigate the underlying mechanism of marital satisfaction that impacts the child’s depression and anxiety through the child’s perceived family functioning and how parent–child co-viewing may interact with the marriage system, bringing change to the family functioning and eventually decreasing depression and anxiety.

## 2. Literature Review

### 2.1. Marital Satisfaction and Family Functioning

Various empirical research has identified the positive association between marital satisfaction and family functionality that happy couples are more likely to function well [10], such as in parenting [11] and solving problems [12]. There might be plausible reasons for the relationship between marital satisfaction and family functioning [3]. For instance, family members exert a reciprocal effect because they are interdependent in nature. Specifically, in the family system, marital discord is related to maladaptive interactions that may deteriorate family functioning, while a satisfying marriage is associated with responsive and sensitive interactions that may facilitate family functioning [11].

Whereas previous studies have identified a strong association between marital satisfaction and the parents’ perception of family functioning, the child’s perception of family functioning is substantially neglected. In fact, children are the ongoing observers of marital interactions, tending to be highly attuned to signals of hostility and affection between the parents and even react to mediate when conflicts occur [13]. Lasting hostility because of marital dissatisfaction will inevitably decrease the child’s perceived family functioning. Meanwhile, as the child’s depression and anxiety are the focal concern of our study, from the perspective of children adjustment, whether the family is perceived to be trustworthy and well-functioned by the children seems more predictive than that parents. Thus, we nuanced our research by first focusing on the association between parents’ marital satisfaction and the child’s perceived family functioning. 

Additionally, most of the studies, collapsing paternal marital satisfaction evaluation with maternal marital satisfaction evaluation, use a combined estimate of the couple as the indicator of marital satisfaction. This composite indicator may provide a distorted view because (1) maternal and paternal marital satisfaction might not always converge; (2) the patterns for marital satisfaction to family functioning may hold differently for the father and mother. For instance, previous research hints that the father’s involvement in the family was more vulnerable to marital satisfaction [14,15] than the mother and that the father tends to interact negatively with the child when they are less happy about marriage [16]. However, another research indicated that mothers who experienced marital dissatisfaction were less involved with their children [17]. Due to the inconsistency, we nuanced the research by using the independent reports of maternal marital satisfaction and paternal marital satisfaction and investigating its respective association with the child’s perceived family functioning. Specifically, we assumed that:

**Hypothesis 1a** **(H1a).** *Paternal marital satisfaction will be positively associated with the child’s perceived family functioning.*

**Hypothesis 1b** **(H1b).** *Maternal marital satisfaction will be positively associated with the child’s perceived family functioning.*

### 2.2. Child’s Perceived Family Functioning and Their Depression and Anxiety

Family functioning is conceptualized as the dynamics that family members care for each other, communicate, interact, solve problems, make decisions, and maintain relationships [18]. Despite the fact that operationalization and the measurements of family functioning do not always converge, the majority of research has documented a negative association between family functioning and various indicators of children’s mental health [5]. That is, children in the better-functioned family reported fewer mental health problems [19], whereas children in the poorer functioned family reported a variety of mental health problems. For instance, Katz and Low indicated that the indicators of family dysfunction, such as fragmented family interactions and negative conflicts, are positively associated with children’s anxiety and depression [20].

Despite the consistent findings on the association between family functioning and a child’s depression and anxiety, most of the research uses parents’ reports of family functioning. However, the child’s perceived family functioning may differ from parents’. In fact, Faust and colleagues [21] indicated that a child’s observations of family dynamics, such as family conflicts and cohesion, are different from the observation of mothers. Compared with the parents’ perception of family functioning, a child’s perceptions of family functioning should be the more proximal predictor of the child’s depression or anxiety levels. Particularly, longitudinal research has stressed that family functioning perceived by the children, relative to family functioning perceived by the parents, was more consistent and had a stronger association with various indicators of children’s psychological well-being [22], i.e., hopelessness, life satisfaction, self-esteem. In a similar vein, children who perceive their family as cohesive, well-adapted, and capable of solving problems are less likely to become anxious or depressed because they are confident in their family. 

In contrast, children who perceive their family as hostile, poorly-adapted, and incapable of solving problems are more likely to become anxious and depressed because they may experience higher levels of stress and helplessness. This reasoning can be supported by empirical studies which reported the negative association between perceived family functioning and the child’s self-injury behaviors, which were highly related to depression and anxiety [23]. Taken together, the following hypothesis is proposed:

**Hypothesis 2a** **(H2a).** *The child’s perceived family functioning will be negatively associated with the child’s anxiety level.*

**Hypothesis 2b** **(H2b).** *The child’s perceived family functioning will be negatively associated with the child’s depression level.*

### 2.3. The Mediating Role of Child’s Perceived Family Functioning

Drawing on the preliminary research about the positive association between maternal/paternal marital satisfaction and family functioning [24] and the negative association between family functioning and various indicators of mental health problems [5], we further expected a mediation association among these focal constructs. Research has shown the direct effect of maternal/paternal marital satisfaction on children’s mental health indicators [5,24] and hints at the possibility of indirect effects through family functioning. In fact, a recent review has indicated the consistent findings that family functioning plays the mediating role between adverse childhood experiences (e.g., neglect) and depression [5]. Parents who are dissatisfied with their marriage were less involved with child-rearing [17], which may, to some extent, lead to neglect. Mainly, children observe the interactions and regulations of their parents [3], forming perceptions of how their family can function, which may, in turn, affect their mental health. Taken together, we assume that marital satisfaction is positively associated with the child’s perceived family functioning, which in turn, is related to lower levels of children’s anxiety and depression. Specially, we assume that,

**Hypothesis 3a** **(H3a).** *Paternal marital satisfaction is positively associated with the child’s perceived family functioning, which in turn, is related to lower levels of the child’s anxiety.*

**Hypothesis 3b** **(H3b).** *Paternal marital satisfaction is positively associated with the child’s perceived family functioning, which in turn, is related to lower levels of the child’s depression.*

**Hypothesis 3c** **(H3c).** *Maternal marital satisfaction is positively associated with the child’s perceived family functioning, which in turn, is related to lower levels of the child’s anxiety.*

**Hypothesis 3d** **(H3d).** *Maternal marital satisfaction is positively associated with the child’s perceived family functioning, which in turn, is related to lower levels of the child’s depression.*

### 2.4. Parent-Child Co-Viewing Moderate the Association between Marital Satisfaction and Family Functioning

Parent–child (TV) co-viewing, among other types of media co-use, is frequently observed in US families [9]. Parent–child media co-use, in general, is the first conceptualized strategy for the parents’ positive intervention in children’s media use [25]. Specifically, co-viewing was defined as “the extent to which parents use media together with their children, without actively engaging in discussions” [26]. Parent–child media co-use is not only related to less negative consequences of child’s independent media use, but also related to more positive parent–child interactions. For instance, parent–child co-using tablets were associated with few parent–child conflicts [27]. In addition, parent–child co-viewing is believed to increase parent–child bonding and facilitate the relationship because parents show their willingness to engage in children’s activities [28]. From the children’s perspective, they may perceive that their parents approved what they are co-viewing [29]. In fact, children reported higher enjoyment of the content when parents viewed programs together with them [30].

Based on the positive effect of parent–child co-viewing, we believe such co-viewing is likely to enhance the positive association between marital satisfaction and children’s perceived family functioning. In addition, family systems stress the interactions among family members and prior studies have long called for multiple perspectives from different family members [31]. We further nuanced the research by investigating the dyadic effect of parent–child co-viewing on the spouses and marital satisfaction on the child’s perceived family functioning. Specifically, we believe that one parent’s marital satisfaction is positively related to the child’s perceived family functioning. Meanwhile, the other parent’s co-viewing is likely to enhance this positive effect. Previous research has revealed that father and mother interactions impact the child. The strengthening pattern will be highly likely if one supportive parent works together with the other supportive parent [32].

In addition, research showed that shared activities among family members are the most important force in developing cohesive relationships and facilitating family functioning through strengthening identity and increasing bonding [31]. Empirical evidence supports this reasoning that media co-use serves as a function of parental availability in parental–child interactions [9]. Moreover, Padilla-Walker et al. [33] found that parent–child co-viewing is associated with higher levels of family connection. Research has verified the positive effect of parent–child co-viewing on enhancing identity and facilitating bonding. We believe that one parent co-viewing may work as an additional impact to facilitate family functioning aside from the positive effect of the other parent satisfying the marriage.

Taken together, we assume that one parent co-viewing will enhance the association between the other parent’s marital satisfaction and the children’s perceived family functioning. We proposed that,

**Hypothesis 4a** **(H4a).** *Paternal co-viewing will enhance the positive association between maternal marriage satisfaction and the child’s perception of family functioning and then decrease the child’s anxiety.*

**Hypothesis 4b** **(H4b).** *Paternal co-viewing will enhance the positive association between maternal marriage satisfaction and the child’s perception of family functioning and then decrease the child’s depression.*

**Hypothesis 4c** **(H4c).** *Maternal co-viewing will enhance the positive association between paternal marriage satisfaction and the child’s perception of family functioning and then decrease the child’s anxiety.*

**Hypothesis 4d** **(H4d).** *Maternal co-viewing will enhance the positive association between paternal marriage satisfaction and the child’s perception of family functioning and then decrease the child’s depression.*

### 2.5. Methods

The study relies on a paper-and-pencil questionnaire distributed in 2020 to a convenience sample of 425 Chinese households. Researchers contacted three primary schools in Beijing, inviting children from Grade 2 to Grade 5 and their parents to participate in the research. Children were invited within the class unit, which was selected randomly in each Grade. The questionnaires were specifically tailored for each member (i.e., mother, father, and children). Questionnaires for the children were distributed by the headteachers of each class and filled by the children during the class. Questionnaires for the parents were taken home and sent back to school by the children the next day. Among all the households, 107 households (25.18%) have one parent, or both parents failed to fill out the questionnaire. Due to these missing values, 318 families were kept for analysis. The children were roughly evenly distributed in gender (52% males), with ages ranging from 8 to 13 (M_age_ = 10.54, SD = 0.97).

### 2.6. Measures

Marital satisfaction [34]. Marital satisfaction was measured with the seven-item Dyadic Adjustment Scale (DAS-7). The items were measured on a six-point Likert scale (0 = always disagree or extremely unhappy; 5 = always agree or perfect), showing a good reliability in mothers (M = 2.90, SD = 0.91, α = 0.84) and fathers (M = 2.94, SD = 0.92, α = 0.83). Sample items include “indicate below the approximate extent agreement or disagreement between you and your partner on aims, goals, and things believed important” and “indicate which best describes the degree of happiness, all things considered, of your relationship.”

Child perceived family functioning [35]. The Family Assessment Device (FAD) was used to assess family functioning. Six questions were asked on a four-point Likert scale (1 = strongly disagree; 4 = strongly agree). Sample items include “We are able to make decisions about how to solve problems” and “In times of crisis, we can turn to each other for support”. The scale research showed satisfactory reliability (M = 3.33, SD = 0.62, α = 0.85).

Parent co-viewing. A modified sub-scale based on the work of Valkenburg et al. (1999) was adapted to assess the frequency of parents’ co-viewing television with their child. In the instructions, we define co-viewing as follows: “The viewing can refer to viewing various programs together with your child from common television, smart television, tablets, and smartphones.” The co-viewing subscale has five items, using a five-point Likert scale (0 = never; 4 = always). Sample items includes “how often does your child watch TV together with you because of a common interest” and “how often does your child laugh together with you about something fun you see on TV?” The average scale score was 3.14 (SD = 0.94, α = 0.86) among fathers and 3.19 (SD = 0.92, α = 0.87) among mothers.

Child’s Anxiety and Depression [36]. Twenties-five items based on Ebesutani et al.’s work was used to assess the child’s mental health, measured on a four-point Likert scale (1 = never; 4 = always). The CAD scale has two sub-scales, an anxiety scale with 15 items and depression with 10 items. Sample items of the anxiety sub-scale include “I worry that something awful will happen to someone in my family” and “I have to do some things in just the right way to stop bad things from happening.” The average scale score was 0.82 (SD = 0.57, α = 0.88). Sample items of the depression sub-scale include “I don’t have energy for things” and “I feel worthless.” The average scale score was 57 (SD = 0.52, α = 0.85).

### 2.7. Analytical Plan

Descriptive statistics and zero-order correlations were conducted using SPSS 24.0. We used the PROCESS macro model 4 and model 7 for SPSS for the mediation model and moderated mediation analysis [37].

## 3. Results

### 3.1. Preliminary Analysis

Table 1 present descriptive statistics and bivariate correlations among the variables. The child’s anxiety and depression were meaningfully associated with parental marital satisfaction. Specifically, paternal marital satisfaction was negatively related to the child’s anxiety level (r = −0.11, *p* = 0.043), whereas maternal marital satisfaction was negatively related to the child’s depression level (r = −0.11, *p* = 0.033). The child’s perceived family functioning was positively related to maternal marital satisfaction (r = 0.13, *p* = 0.014) but not to paternal marital satisfaction (r = 0.05, *p* = 0.391). Additionally, the child’s perceived family functioning was negatively related to the child’s anxiety (r = −0.29, *p* < 0.001) and depression (r = −0.38, *p* < 0.001). These correlations provided the initial evidence for mediating the chain among maternal marital satisfaction, the child’s perceived family functioning, and the child’s depression. However, due to the insignificance association between paternal marital satisfaction and the child’s perceived family functioning (r = 0.05, *p* = 0.391), we did not conduct the mediation model with paternal marital satisfaction.

**Figure 1:**
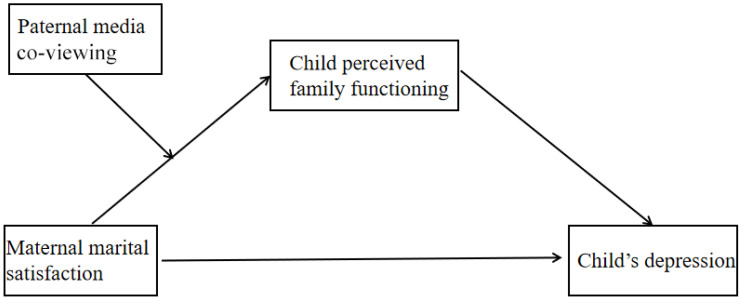
The moderated mediation model.

Specifically, we ran two models separately. First, we ran a mediation model: The child’s depression was entered as the dependent variable, maternal marital satisfaction as the independent variable, and the child’s perceived family functioning as a mediator. Second, we run a moderated-mediation model: The child’s depression was entered as the dependent variable, maternal marital satisfaction as the independent variable, the child’s perceived family functioning as the mediator, and paternal co-viewing as the moderator, with maternal co-viewing as the covariate (see Figure 1).

### 3.2. The Mediation Model

The results for model 4 showed that maternal marital satisfaction was positively associated with the child’s perceived family functioning (b = 0.087, se = 0.035, *p* = 0.014, 95% CI [0.018, 0.156]). The child’s perceived family functioning was negatively associated with the child’s depression (b = −0.320, se = 0.042, *p* < 0.001, 95% CI [−0.402, −0.238]). The child’s perceived family functioning mediated the association between maternal marital satisfaction and the child’s depression (indirect effect = −0.028, bootstrap SE = 0.013, 95% CI [−0.055, −0.004]).

### 3.3. The Moderated-Mediation Model

**Figure 2:**
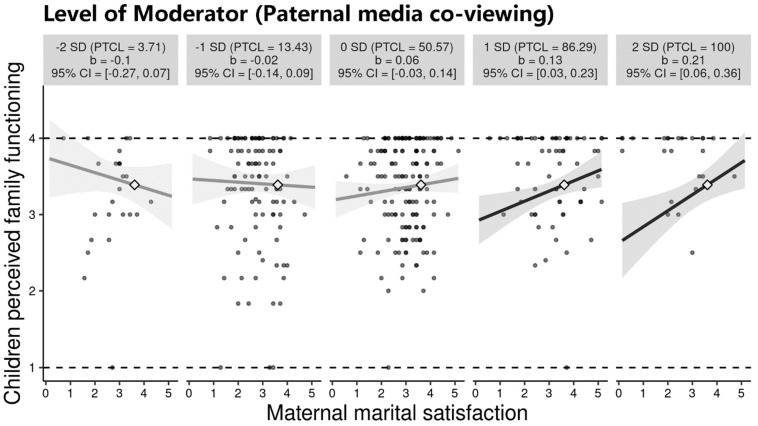
The moderated plot. Note. The small multiples illustrated the interaction across the range from 2 SD below to 2 SD above the mean of paternal co-viewing [38]. Each graphic showed the computed 95% confidence region (shaded area), the observed data (gray circles), and the maximum and minimum values of the outcome (dashed horizontal lines). CI = confidence interval; PTCL = percentile.

The results for moderated-mediation (model 7) showed that, after controlling for maternal co-viewing, paternal co-viewing moderated the association between maternal marital satisfaction and the child’s perceived family functioning, and then affected child’s depression (index = −0.029, bootstrap SE = 0.014, 95% CI [−0.059, −0.002]). See Table 2 for the full results. As depicted in Figure 2, the moderation analysis showed a significantly stronger relationship between maternal marital satisfaction and the child’s perceived family functioning at higher levels of paternal co-viewing. In fact, when paternal co-viewing is below the average value (M = 3.14), meaning that the father reported co-viewing less frequently than “sometimes”, the association between maternal marital satisfaction and the child’s perceived family functioning is not significant. When paternal co-viewing is around and above one standardized deviation than the average value, the association between maternal marital satisfaction and the child’s perceived family functioning is significantly positive. The higher levels of paternal co-viewing, the stronger such positive association will be.

Taken together, H1a was not supported that paternal marital satisfaction was not related to the child’s perceived family functioning, whereas H1b was supported that maternal marital satisfaction was positively associated with the child’s perceived family functioning. H2a and H2b were both supported that the child’s perceived family functioning was negatively associated with the child’s anxiety and depression. Regarding the four mediation hypotheses (H3a–H3d), only H3d was supported that maternal marital satisfaction was positively associated with the child’s perceived family functioning, which, in turn, was negatively related to the child’s depression. Finally, among four moderated-mediation hypotheses (H4a–H4d), only 4d was supported that paternal co-viewing enhanced the positive association between maternal marital satisfaction and the child’s perceived family functioning and then decreased the child’s depression.

## 4. Discussion

Despite a large body of research concerning how family functioning affects the child’s mental health, in the digital age, parent–child media co-use has offered opportunities for improving family functioning; however, the joint effect of family dynamics and media co-use is largely unknown. The current study thus attempted to take an integrated look at how parental marriage satisfaction is associated with the child’s perceived family functioning, which, in turn, affects the child’s anxiety and depression. Specially, we investigated whether paternal co-viewing can facilitate the positive effect of maternal marital satisfaction on the child’s perceived family functioning and then impact the child’s anxiety and depression. In a similar vein, we also investigated the symmetrical enhancing effect of maternal co-viewing on the positive impact of paternal marital satisfaction and the child’s perceived family functioning, which, in turn, impact the child’s anxiety and depression.

Partially supporting H1, the results showed that only maternal marital satisfaction is positively associated with the child’s perceived family functioning, meaning that the happier the mom about her marriage, the better functioning her child perceived about the family. On the contrary, whether the father feels satisfied with marriage seems not significantly related to the child’s perception of family functioning. This finding might be explained that the impact of marital satisfaction on the mother’s involvement in the family is greater than the father’s, especially for children rearing. Consequently, whether the mother is happy about their marriage will bring a larger influence on the child’s perceived family functioning than the father. Previous empirical research can support this reasoning that the association between marital satisfaction and parent–child qualified engagement is significant for mothers but not for fathers [17]. Our research thus substantiates the underpinning and the volatile role of the mother in shaping family dynamics that a happy wife is essential for the family to function well.

Supporting H2, the child’s perceived family functioning is negatively associated with the child’s levels of anxiety and depression. Moreover, the child’s perceived family functioning partly explained the indirect association between maternal marital satisfaction and the child’s depression, which supported H3d. We did not conduct the mediation analysis due to the insignificant association between paternal marital satisfaction and the child’s perceived family functioning and maternal marital satisfaction and the child’s anxiety. It is worth noting that the correlation coefficients for marital satisfaction with child’s anxiety and depression hold slightly differently for mothers and fathers, with maternal marital satisfaction more strongly related to depression and paternal marital satisfaction more strongly to anxiety. These findings hint that happily married mothers may provide a sense of safety or certainty as a higher maternal marital satisfaction is related to a lower level of child’s depression. In contrast, happily married fathers may provide more of a sense of confidence as higher paternal marital satisfaction is related to lower levels of child anxiety.

Regarding the four mediation hypotheses, the only supported hypothesis is that maternal satisfaction increases the child’s perceived family functioning, which, in turn, decreases the child’s levels of depression. Besides, paternal co-viewing interacted with maternal marital satisfaction to impact the child’s perceived family functioning and then impact the child’s depression. That is, fathers who spend more time co-viewing programs with their child facilitate the positive effect of happily married mothers on the child’s perceived family functioning and then decrease the child’s depression levels.

### 4.1. Theoretical Implications and Practical Implications

The current research suggests several new conclusions about the integrated association between the happiness of parents about marriage, the child’s perception of their family functioning, co-parenting with media engagement, and the child’s mental health. The findings are especially noteworthy because they are based on the independent assessment of parents’ co-viewing, marital satisfaction, and the child’s mental health. This nuanced methodology warrants the reliability of association among the aforementioned focal constructs as the common method bias was, to some extent, controlled. In addition, these findings contribute to the family system theory by incorporating parent–child co-viewing to facilitate family member interactions and functionality. This is especially relevant since various media use has become the fixture of the family system and previous research suggested its potential to generate changing family dynamics [39]. The current research thus provided the initial evidence to support this rationale from a family system perspective. Practically, as evidence suggests that paternal co-viewing can enhance the positive effect of maternal marital satisfaction and then decrease the child’s depression, interventions could therefore be promoted to encourage father–child co-viewing via various devices in daily interactions. In this way, co-viewing can function as an agent to mobilize more family members and add an additional crossover effect to improve the child’s mental health.

### 4.2. Limitations and Suggestions for Future Directions

The findings have to be interpreted with several limitations. First, although the research proposed a mediation effect, one should keep in mind that the correlational nature of the design that interpretations cannot assume causality. Future studies may consider using a longitudinal design to investigate the causal effect and test the long-term effect of co-viewing. Although the current study theorized parental co-viewing as the moderator between parental marital satisfaction and the child’s perceived family functioning, other competitive models with parental co-viewing as the mediator between parental marital satisfaction and the child’s perceived family functioning are also likely. Longitudinal data was also required to test these models. Second, the study only includes co-viewing as the indicator of parent–child media use, which refers to the joint engagement of media use without active parental mediation. However, parents are likely to engage in restrictive mediation and instructive mediation due to the concerns about the negative effects of media use [27]. As such, the current design refrains from investigating the joint effects of restrictive and instructive co-viewing in parent–child media interactions. In addition, we focus on co-viewing programs across various devices; however, other types of media co-use (e.g., co-playing video games) were not investigated. This refrains from generalizing the findings to other types of media co-use. For instance, co-playing video games involve more cooperative interactions that might be more effective in facilitating the child’s perception of family functioning. A future study is called for to take integrated but also nuanced measurements of parent–child media interactions to provide a more fine-grained picture of how these interactions may bring change to family functionality and the child’s depression and anxiety. Third, we deliberately limited the age of participants (i.e., children younger than 8-years-old) due to the complexity of the questionnaire, so not all members have a voice. We do not have the data for families with more than one child. However, families with more than one child may change parent–child (media co-use) dynamics. Fourth, roughly 25% of parents failed to respond to our research. Although it happens that one or even both parents were not available during that one night, the probability that the parents who responded are more satisfied with their marriage than those who did not respond cannot be ignored. As such, the interpretation of the results should be considered with this possible bias. Finally, the self-reported data we use could inevitably induce recall bias.

## 5. Conclusions

Children with happily married mothers are likely to perceive their family functioning well, and such perception may decrease their depression levels. In contrast, paternal marital satisfaction may not directly impact the child’s perception of family functioning. Fathers, however, can promote the child’s perception of family functioning by co-viewing programs together with their children, and jointly with happily married mothers, children will be less likely to feel depressed.

## Figures and Tables

**Table 1:** Mean, standard deviation, and zero-order correlations (N = 318).

Variable	M	SD	1	2	3	4	5	6
1. Paternal marital satisfaction	2.94	0.92	1					
2. Maternal marital satisfaction	2.90	0.91	0.78 ***	1				
3. Paternal co-viewing	3.14	0.94	0.35 ***	0.25 ***	1			
4. Maternal co-viewing	3.19	0.91	0.05	0.26 ***	0.68 ***	1		
5. Child’s perceived family functioning	0.82	0.57	0.10 *	0.13 *	0.04	0.09	1	
6. Child’s anxiety	0.57	0.52	−0.11 *	−0.09	0.05	0.01	−0.29 ***	1
7. Child’s depression	3.33	0.62	−0.09	−0.11 *	−0.03	0.01	−0.38 ***	0.76 ***

Note. * *p* < 0.05, *** *p* < 0.001.

**Table 2:** Results of moderated mediation analysis (N = 318).

	Child’s Perceived Family Functioning				Depression			
Predictors	*B*	SE	LLCI	ULCI	*B*	SE	LLCI	ULCI
Constant	3.91	0.41	3.10	4.72	1.75	0.18	1.40	2.11
Maternal marital satisfaction	−0.20	0.13	−0.46	0.06	−0.06	0.03	−0.12	0.01
Child’s perceived family functioning					−0.35	0.04	−0.44	−0.27
Paternal co-viewing	−0.20	0.13	−0.46	0.06				
Paternal co-viewing × Child’s perceived family functioning	0.08	0.04	0.01	0.16				
*R* ^2^	0.03 *				0.18 ***			
Conditional indirect effects of maternal marital satisfaction on depression at a value of paternal co-viewing through children perceived family functioning								
Paternal co-viewing	Effect		Boot SE		Boot LLCI		Boot ULCI	
−1 SD	0.007		0.022		−0.037		0.051	
M	−0.020		0.017		−0.055		0.011	
+1 SD	−0.048		0.021		−0.091		−0.011	
Index of moderated mediation mediator	Index		Boot SE		Boot LLCI		Boot ULCI	
	−0.029		0.014		−0.059		−0.002	

Note. Significance testing for indirect effects based on 95% confidence intervals (CIs) using 5000 bootstrap sample. SE: standard error. All models were controlled for maternal media co-viewing. * *p* < 0.05, *** *p* < 0.001.

## Data Availability

The data presented in this study are available on request from the corresponding author.
